# Sexting: Prevalence, Predictors, and Associated Sexual Risk Behaviors among Postsecondary School Young People in Ibadan, Nigeria

**DOI:** 10.3389/fpubh.2017.00096

**Published:** 2017-05-08

**Authors:** Oluwatoyin Olatunde, Folusho Balogun

**Affiliations:** ^1^Institute of Child Health, College of Medicine, University of Ibadan, Ibadan, Nigeria; ^2^University College Hospital, Ibadan, Nigeria

**Keywords:** sexting, postsecondary schools, sexual risk behaviors, problematic phone use, young people

## Abstract

**Background and aims:**

Sending and receiving sexually suggestive or explicit images or texts (sexting) have been shown to be associated with health risk behaviors but literature about this phenomenon is scarce in Nigeria. This study looked at the prevalence, predictors, and associated sexual risk behaviors of sexting among postsecondary school young persons in Ibadan, Nigeria.

**Methods:**

This was a cross-sectional study using a self-administered questionnaire. Data were obtained for sociodemographic characteristics and sexual orientation, sexting behavior, personality assessment (using the International Personality Item Pool Big-Five factor markers), indicators for problematic phone use (using Mobile Phone Problem Use Scale), and sexual behavior. Chi square test and logistic regression were used for data analysis with *p* = 0.05.

**Results:**

Five hundred seventy-five participants were recruited, age range 14–24 years, and 46.0% were males. Twenty percent had sent sexts, while 33.2% had received sexts. Fifty-four percent had high scores in extraversion, 46.5% had moderate–severe problematic phone use. Sixteen percent had ever had sex, and 40.0% of these had multiple sexual partners. Males were more likely than females to have sent sexts (OR = 2.67, 95% CI: 1.68–4.24). Having a high extraversion score (OR = 2.44, 95% CI: 1.35–4.41) and moderate–severe problematic phone use (OR = 5.56, 95% CI: 2.73–11.32) was predictive of sexting. Sending and receiving of sext were significantly associated with ever having sexual intercourse (OR = 4.01, 95% CI: 2.25–7.17 and OR = 2.96, 95% CI: 1.72–5.12, respectively).

**Conclusion:**

Sexting was prevalent among postsecondary school young persons in Ibadan and was associated with male sex and problematic phone use. Intervention targeted at the identified susceptible group of young people may reduce its associated problems in this study group.

## Introduction

Sexting is the act of using mobile phones or electronic devices to send and/or receive sexually suggestive and or sexually explicit images or texts (1, 2). It is a relatively new phenomenon among young people (2). There is a great variation in the prevalence of sexting among young people as reported in the literature. This variation can be explained by the different methodologies employed and the different definitions given to sexting in these studies (1, 3, 4). Mitchell et al. conducted a phone interview with adolescents and caregivers in a national survey in the United States to investigate sexting and got a prevalence of 9.6% (1). The adolescents in this study may have underreported sexting activity because of the presence of caregivers in the homes despite the efforts made to ensure privacy and confidentiality. On the other hand, Rice et al. reported a prevalence rate of 15.0% among Los Angeles high school adolescents using a self-administered questionnaire in the school environment (3). The adolescents in this case were more open about their involvement in sexting due to the absence of caregivers. Sexting has been reported to be more prevalent among the older age group (5). Sexting has also been defined in different ways, and this resulted in high prevalence rates when the definition is broad to include sending of both sexually suggestive or explicit pictures and texts. Lower prevalences were reported when the definition is limited to sending of just sexually suggestive or explicit pictures or texts. Prevalence reported among young people in the United State varies between 1.0 (1) and 44.0% (6).

Sexting has been described as a harmless behavior and a normal way of communicating intimacy by some scholars (7, 8). This suggestion is strengthened by the fact that sexting has been observed to be common among romantically involved young people (1, 9, 10). Some adolescents also use sexting as a way of expressing their sexuality and serves an alternative to more explicit physical sexual acts (3). Adolescents who engage in sexting have reported that their peers also sext, and this suggests that this activity is being viewed as a normal behavior among them (1, 3).

However, sexting has been shown to be associated with some factors, including health risk behaviors (some of which have devastating consequences) and environmental and personal factors. Some of the health risk behaviors include risky sexual behaviors, pornography and substance use, bullying, and even suicide (11–13). Young people who engage in sexting were more likely to engage in risky physical sexual activities (2, 3, 12) and more likely to seek the fulfilment of the aroused desires shortly after exchanging sexual messages with their sexual partners (11, 12). There has been a suggestion that young people who have online sexual behaviors (which can include sexting) were more likely to have problematic family background (13). This was corroborated by Benotsch et al. who reported that adolescents who live with both parents were less likely to be involved in sexting (6). Young people who get involved in sexting have been reported to develop new risky sexual behaviors (6). Personality has been shown to be a strong predictor of behaviors (14, 15), and personality traits that have been associated with sexting include extraversion, neuroticism, and low agreeableness (16). The negative consequences of sexting may be a serious issue among young people because of their inability to handle complex emotional issues which sometimes accompany sexting. There are in addition, external stressors like academic and social demands which are common at this stage of development.

There has been a great increase in the rate of phone use in developing countries including sub-Saharan Africa in the last decade (17, 18). This has also increased smart cell phone possession by young people in this region (19), even among those of lower socioeconomic status (17). These phones have become more affordable and have an increasing complex capability to take photos, create videos, and connect to social networking sites (4, 18). Thus, with increasing possession of smart phones and access to the Internet, it is possible that sexting occurs among young people in Nigeria and other developing countries just as it is found in developed countries. However, literature about this phenomenon barely exists in Africa, including Nigeria. It is, therefore, important to determine the pattern of sexting and its associated problems among Nigerian young people as there may be sociocultural influences, which may make the pattern different from what has been reported earlier in the literature among young people from other regions. Thus, this study was conducted to determine the prevalence of sexting, its predictors, and associated sexual risk behaviors among postsecondary school young people attending Advanced Education centers and pre-varsity examination preparatory centers in Ibadan, Nigeria.

## Materials and Methods

The study was conducted in Ibadan, the capital city of Oyo State, which is the second largest state in Nigeria.

### Study Population

This study was conducted among postsecondary school students attending Advanced Education centers and pre-varsity examination preparatory centers in Ibadan. The postsecondary school study population was purposively selected because they were more likely to have personal phones and be sexually active compared with the younger age group.

### Sample Size Calculation

Sample size was calculated using the formula for cross-sectional studies as described by Kish (20) and sexting prevalence of 20.0%, which was reported from a study in Peru, a middle-income country with similar sociopolitical and economic background like Nigeria (21). The level of precision was set at 5.0%. Putting into consideration the clustering nature of the schools (calculated sample size was multiplied by an effect factor of 2) and a non-response rate of 10.0%, the final sample size was 575 students.

### Study Design and Sampling

A cross-sectional study design was used with two-stage sampling technique employed to select 4 out of 6 registered Advanced Education Centers, 10 out of an estimated 30 pre-varsity exam preparatory centers and 575 students.

### Data Collection Procedure

Sexting in this study was defined as the sending or receiving of nude or almost nude pictures and sexually suggestive pictures or text messages. A pretested, structured, self-administered questionnaire was used to obtain data about sociodemographic characteristics, sexual orientation, sexting prevalence, predictors of sexting (mobile phone access and use, personality traits, and problematic mobile phone use), and sexual risk behaviors from the respondents.

### Data Analysis

Socioeconomic class was determined using the method described by Oyedeji in Nigeria (22) using a composite number obtained from education level and occupation of both parents. In this classification, class I is the highest while class V is the lowest economic class. Personality traits were assessed using the 50-Item set of International Personality Item Pool Big-Five Factor Markers (23) and scored on 5-point Likert scale. Overall score for each trait less than the mean score of the trait indicated low-trait score. Problematic mobile phone use was assessed using the 27-item Mobile Phone Problem Use Scale (24) and scored on 10-point Likert scale. Total scores ≤76 indicated low–moderate degree and scores ≥77 indicated moderate–severe degree of problematic mobile phone use. Respondents were told that they could skip any question they did not feel comfortable with and that they could opt out of the study anytime they want. Data were analyzed using Statistical Package for Social Sciences version 22. Descriptive statistics were reported and categorical variables were compared using Chi square test. Variables which showed significant association with sexting were included in a multivariate logistic regression model. Predictors for each of the different sexting activities were determined, and each of the models were then adjusted for sexual orientation, socioeconomic class, family type, who the respondent live with, and the identified predictors. Sexual behaviors which predicted sending and receiving of sext (both pictures and text) were also determined, and this was then adjusted for sexual orientation, age, sex, and the different sexual behaviors. Significance level for all tests was 5.0%.

## Results

### Sociodemographic and Family Characteristics

Table 1 shows the sociodemographic and family characteristics of the respondents. The mean age of the respondents was 17.4 ± 2.0 years. The majority (82.3%) of the respondents were within the age group 15–19 years. Fifteen percent were lesbian/gay/bisexual/transgender (LGBT). Almost all (96.7%) of the young people interviewed had personal mobile phones. Those who had high personality trait score were 306 (53.8%), 287 (50.1%), 319 (55.9%), 344 (60.5%), and 273 (47.6%) for extraversion, conscientiousness, intellect score, emotional stability, and agreeableness, respectively. Moderate to severe problematic phone use was also found in 265 (46.5%) of the respondents.

**Table 1:** **Sociodemographic and family characteristics of respondents**.

Characteristics	*N* (%)
**Age (years)**	
10–19	472 (86.0)
20–24	77 (14.0)
**Sex**	
Male	259 (46.0)
Female	304 (54.0)
**Sexual orientation**	
Heterosexual	267 (62.5)
Unsure	97 (22.7)
Bisexual	52 (12.2)
Gay/lesbian	8 (1.9)
Transgender	3 (0.1)
**Parental socioeconomic class^a^**	
Class I	157 (27.8)
Class II	328 (58.2)
Class III	70 (12.4)
Class IV	9 (1.6)
Class V	0 (0.0)
**Fathers’ education**	
No formal training	11 (2.1)
Primary	32 (6.2)
Secondary	92 (17.7)
Postsecondary	385 (74.0)
**Mothers’ education**	
No formal training	14 (2.7)
Primary	24 (4.7)
Secondary	101 (19.6)
Postsecondary	376 (73.0)
**Type of current training**	
A levels	260 (46.0)
Post UTME	181 (32.0)
GCE	108 (19.1)
NECO	5 (0.9)
Others	11 (1.9)

*There was non-response to some of the questions*.

*^a^Class I is the highest while Class V is the lowest*.

*NECO, National Examination Council (Nigeria); GCE, General Certificate Examination; UTME, Unified Tertiary Matriculation Examination*.

### Sexting Behavior

Among the respondents, 183 (31.9%) knew someone who had sent sexually suggestive images or text, 115 (20.1%) had sent sexts, and 189 (33.2%) had received sexts. Table 2 shows prevalence of each sexting behavior of the postsecondary school young persons studied. Reasons for sending sext include for fun (57.9%), a response to a request by their partners in a romantic relationship (17.9%), to impress the receivers (14.7%), and to keep an ongoing relationship (5.3%). There were no statistically significant differences in the age, sex, and sexual orientation of the respondents who sent sext and those who did not (*p* = 0.12, *p* = 0.05, and *p* = 0.23, respectively), although a higher percentage of males (11.6 vs 6.9%), those older than 19 years (14.3 vs 8.7%), and LGBT (14.3 vs 10.9%) were sending such messages. Table 3 shows the other sociodemographic characteristics of those who had sent or received sext.

**Table 2:** **Pattern of sexting behavior of respondents**.

Sexting behavior	*N* (%)
**Sent naked/half-naked picture of oneself**	
Yes	52 (9.1)
No	522 (90.9)
**Received naked/half-naked picture**	
Yes	162 (28.4)
No	409 (71.6)
**Sent sexual text messages**	
Yes	96 (16.7)
No	478 (83.3)
**Received sexual text messages**	
Yes	135 (23.6)
No	436 (76.4)
**Asked for naked/half-naked pictures**	
Yes	48 (8.4)
No	523 (91.6)
**People to whom the sext was sent**	
Current boyfriend/girlfriend	12 (23.5)
Ex-boyfriend/girlfriend	14 (27.5)
Friend	18 (35.3)
Stranger	7 (13.7)
**How the sext was sent**	
WhatsApp	26 (57.8)
Others (BBM = 25.0%, 2go = 50%, Snapchat = 25.0%)	9 (20.0)
Facebook	6 (13.3)
Bluetooth	3 (6.7)
MMS	1 (2.2)

*MMS, multimedia service; BBM, BlackBerry Messenger*.

**Table 3:** **Sociodemographic characteristics among respondents who had received or sent sexts**.^a^

Sociodemographics	Ever received sext		*p*-Value	Ever sent sext		*p*-Value
	Yes (%)	No (%)		Yes (%)	No (%)	
**Age (years)**						
10–19 (*n* = 472)	26.9	73.1	0.03	8.7	91.3	0.120
20–24 (*n* = 77)	39.0	61.0		14.3	85.7	
**Sex**						
Male (*n* = 259)	33.6	66.4	0.01	11.6	88.4	0.054
Female (*n* = 304)	23.9	76.1		6.9	93.1	
**Respondents’ marital status**						
Single (*n* = 569)	28.1	71.9	0.01	9.0	91.0	0.143
Co-habiting (*n* = 3)	100.0	0.0		33.3	66.7	
**Respondents’ sexual orientation**						
Heterosexual (*n* = 267)	34.1	65.9	0.03	10.9	89.1	
Other sexual orientation (LGBT) (*n* = 63)	36.5	63.5		14.3	85.7	0.229
Unsure of sexual orientation (*n* = 97)	20.6	79.4		6.2	93.8	

*There was non-response to some of the questions*.

*^a^Chi square test used in analysis*.

*LGBT, lesbian, gay, bisexual, transgender*.

### Sexting Predictors

Table 4 shows moderate to severe problematic phone use was a predictor for both sending of sexually suggestive pictures and sexually suggestive text messages. Other predictors of sending text messages with sex contents include age older than 19 years, male sex, and high extraversion personality. While high extraversion personality (OR: 2.03; 95% CI = 1.24–3.31; *p* = 0.01) and those who had moderate–severe problematic phone use (OR: 2.36; 95% CI = 1.47–3.81, *p* < 0.01) were more likely to have received sexts.

**Table 4:** **Predictors of respondents’ sexting behaviors**.^a^

	Unadjusted estimates			Adjusted estimates^b^		
	OR	95% CI	*p*	OR	95% CI	*p*
**Sent naked/half-naked pictures**						
**Problematic phone use**						
Low–moderate problematic phone use (*n* = 305)	1	–		1	–	
Moderate–severe problematic cell phone use (*n* = 265)	4.69	2.16, 10.17	<0.01	5.56	2.73, 11.32	<0.01
**Sent sexual texts**						
**Age**						
≤19 years (*n* = 472)	1	–		1	–	
20–24 years (*n* = 77)	2.31	1.23, 4.33	<0.01	2.15	1.23, 3.73	<0.01
**Sex**						
Female (*n* = 304)	1	–		1	–	
Male (*n* = 259)	1.91	1.14, 3.22	0.02	2.67	1.68, 4.24	<0.01
**Extraversion**						
Low (*n* = 263)	1	–		1	–	
High (*n* = 306)	2.09	1.22, 3.58	0.01	2.44	1.35, 4.41	<0.01
Problematic phone use						
Low–moderate problematic phone use (*n* = 305)	1	–		1	–	
Moderate–severe problematic phone use (*n* = 265)	2.60	1.52, 4.45	<0.01	2.61	1.48, 4.60	<0.01
**Received naked/half-naked pictures**						
**Extraversion**						
Low (*n* = 263)	1	–		1	–	
High (*n* = 306)	2.03	1.24, 3.31	0.01	1.54	1.05, 2.27	0.03
**Problematic cell phone use**						
Low–moderate problematic phone use (*n* = 305)	1	–		1	–	
Moderate–severe problematic phone use (*n* = 265)	2.36	1.47, 3.80	<0.01	2.37	1.53, 3.67	<0.01
**Received sexual text**						
**Age**						
≤19 years (*n* = 472)	1	–		1	–	
20–24 years (*n* = 77)	1.97	1.07, 3.62	0.03	1.17	0.60, 2.26	0.65
**Type of current training**						
Other types of training (*n* = 305)	1	–		1	–	
A levels (*n* = 260)	1.63	1.02, 2.60	0.04	1.81	1.31, 2.90	0.01
**Extraversion**						
Low (*n* = 263)	1	–		1	–	
High (*n* = 306)	1.66	1.05, 2.66	0.03	1.46	0.32, 0.84	0.01
**Problematic cell phone use**						
Low–moderate problematic phone use (*n* = 305)	1	–		1	–	
Moderate–severe problematic phone use (*n* = 265)	2.55	1.60, 4.06	<0.01	2.35	1.47, 3.77	<0.01
**Asked for naked/half-naked pictures**						
**Father’s education**						
Other class of father’s education (*n* = 139)	1	–		1	–	
Father with postsecondary education (*n* = 376)	4.61	1.41, 15.14	0.01	0.40	0.15, 1.05	0.06
**Problematic cell phone use**						
Low–moderate problematic phone use (*n* = 305)	1	–		1	–	
Moderate–severe problematic phone use (*n* = 265)	6.48	2.36, 17.77	<0.01	7.98	3.22, 19.78	<0.01

*^a^Only significant predictors are shown*.

*^b^Each model was adjusted for sexual orientation, socioeconomic class, family type, who the respondent lives with, and the identified predictors*.

### Sexting and Associated Sexual Risk Behaviors of Respondents

Ninety (16.0%) respondents had ever had sex and significantly, 30 (33.3%) of them were older than 19 years. Early sexual debut (initiating sex before or at 14 years of age) was reported by 15 (16.7%) of them. Only 48 (53.3%) used condom at the last sexual intercourse, and 36 (40.0%) had multiple sexual partners. Alcohol or drugs were used at the last sexual intercourse by 18 (20.0%) of them. Table 5 shows the relationship between sexting and sexual risk behaviors. Among the respondents, 15.4% reported that they had sexual intercourse for the first time with someone after they had sent or received sexual pictures or text messages.

**Table 5:** **Relationship between sexting and respondents’ sexual risk behaviors**.^a^

	Unadjusted estimates		Adjusted estimates^b^	
	OR	95% CI	OR	95% CI
**Sent sext**				
**Ever had sex**						
No (*n* = 90)	1	–	1	–
Yes (*n* = 472)	5.08	3.12, 8.27	4.01	2.25, 7.17
**Early sexual debut**						
>14 years (*n* = 75)	1	–	1	–
≤14 years (*n* = 15)	1.40	0.52, 3.74	0.45	0.12, 1.70
**Multiple sexual partners**						
No (*n* = 54)	1	–	1	–
Yes (*n* = 36)	1.50	0.59, 3.83	1.35	0.41, 4.48
**Condom use at the last sex**						
Yes (*n* = 48)	1	–	1	–
No (*n* = 42)	2.13	0.91, 4.98	1.32	0.41, 4.23
**Sex under the influence of drug/alcohol**						
No (*n* = 72)	1	–	1	–
Yes (*n* = 18)	0.68	0.23, 1.97	0.38	0.10, 1.48
**Received sext**				
**Ever had sex**						
No (*n* = 90)	1	–	1	–
Yes (*n* = 472)	3.70	2.32, 5.90	2.96	1.72, 5.12
**Early sexual debut**						
>14 years (*n* = 75)	1	–		1
≤14 years (*n* = 15)	1.29	0.47, 3.56	0.39	0.10, 1.57
**Multiple sexual partners**						
No (*n* = 54)	1	–	1	–
Yes (*n* = 36)	1.67	0.66, 4.18	1.58	0.47, 5.29
**Condom use at the last sex**						
Yes (*n* = 48)	1	–	1	–
No (*n* = 42)	1.12	0.48, 2.61	0.83	0.25, 2.72
**Sex under the influence of drug/alcohol**						
No (*n* = 72)	1	–	1	–
Yes (*n* = 18)	0.62	0.22, 1.72	0.37	0.10, 1.44

*^a^Apart from ever had sex, the other results are for only sexually active respondents*.

*^b^Each model was adjusted for sexual orientation, age, sex and the different sexual behaviors*.

## Discussion

Among our study population, there were young people involved in sexting, and this was significantly associated with high extraversion personality trait, moderate to severe problematic phone use, and some risky sexual behaviors. The prevalence of sexting was comparable with both the finding from Peru (21) (which is a developing country just like Nigeria but with some sociocultural differences) and from South Africa (25). This prevalence was, however, higher than that reported from another study in the United States (3). All these studies used the broad definition of sexting as used in the present study, which is sending of sexually explicit or suggestive texts or images. However, the present study included an older age group as it looked at young people with age up to 24 years, while the Peruvian and American studies looked at adolescents aged 12–18 years and the South African study had adolescents aged between 12 and 19 years. Therefore, the sexting prevalence may be higher in Peru if a similar age group was studied as sexting has been associated with increasing age in America and South Africa (12, 25). However, the prevalence of sexting among populations of young people from developed countries appeared to be higher even among the younger age groups when sexting was given a broad definition as done in this study (2, 12). This may be because the Internet access is more readily available and cheaper in these regions. The total absence of young people in socioeconomic class V is likely due to the low parental education and less skilled occupation which is not likely to encourage higher education pursuit of their children, and so, such young people are not likely to be present in the study population.

It is interesting to note that 14.8% of the respondents were LGBT in a country were such sexual acts are punishable by law. There may actually be an underreporting of these sexual orientation as LGBT is not socially or legally acceptable in Nigeria, and if caught, they would be imprisoned. However, these people form an important group as they tend to have special health needs (26, 27).

There were more receivers of sexts in this study, as reported in earlier studies (12), than senders. This may be attributed to multiple recipients from one sender as in the case of blackmail or the possibility of respondents receiving sexts from older adult senders, whose age group was not captured by the study. This may constitute a threat in form of sexual molestation or exploitation of these young people by the older adults.

The reasons given for sending sext suggested that sexting was seen to be important in social relationships, and it was being used for maintenance of ongoing relationships and probably, to start new ones, by impressing people around them. This has been noted in earlier studies (11). There is a need for qualitative studies to clearly define the details of the reasons for sending sext including the role of peer pressure. Respondents participated in sexting with persons they knew both on- and offline, but of more concern is the trend of sharing such messages with strangers whom they met online and is not known to them in person (13, 28). Whether this behavior leads to actual physical sexual activity with the stranger is not known as it was not explored in this study. There may also be the dangers of sexual molestation, sexual abuse including rape as the true identity of these strangers is not known. Sending such messages to ex boy/girl friends can also result in blackmail which could also have undesirable outcomes.

Unlike the reports from the United States where sexting was mainly *via* text messaging (1, 29), most of the naked/half-naked pictures in this study were sent through the online mode with the aid of free social networking apps (WhatsApp, 2go, Snapchat, Viber, etc.) available on cell/mobile phones. The low likelihood of text messaging of such images among the study participants in this study may be due to the fact that the service attracts a fee. The preference for the online free social networking applications for sexting among the studied participants may be a result of the advent of affordable smart phones with pre-installed free social networking apps. The availability of cheap, youth friendly mobile data plans that are being competitively marketed by the different mobile network providers and possible craving for social connectivity *via* sharing of photos, texts and videos online may be the drive behind this.

A higher proportion of male respondents were involved in sexting, though the rate was not significantly different from that found among females. This is same as found in the Peruvian study (21) but this finding differs from what had been previously reported from American studies, were females were more likely than males to send sexts, making females more vulnerable to cases of cyberbullying, suicide, and mental problems that had been reported among them (1, 10, 12, 30). This may reflect the low level of societal tolerance of expression of sexuality by young females in the study area. It may also be an underreporting on the part of the female respondents because of cultural expectation that they should not be expressive regarding their sexuality.

The older age group were also more involved in sexting similar to what have been reported earlier in the literature (2, 12), and this may strengthen the fact that sexting is done in the context of romantic relationships. This could be due to older adolescents being more likely to be involved in romantic relationships and be sexually active compared with the younger ones. This may explain the association between sexting and involvement in actual sexual intercourse in this study. It may also be the reason why sexting is sometimes viewed as an expression of affection.

The LGBT group had higher rates of sending and receiving of sext compared with those who were heterosexual even though the proportion sending sext was not significantly different from those who were heterosexual. The LGBT may feel more comfortable and safe expressing their sexuality through virtual means to avoid being arrested or stigmatized. There may be other reasons for this difference which can be explored in future research.

Of the five personality traits assessed, the high extraversion trait strongly predicted both sending and receiving of sext similar to what had been previously reported (16), and this finding may be due to the characteristics of this trait, including being sociable and fun-seeking. This also supports the reasons given for sexting by the respondents and strengthens the suspicion that sexting is seen as a way of socializing by young people. Neuroticism (low-emotional stability) and low-agreeableness traits on the contrary were not found to predict sexting. This finding was different from what was reported by Delevi and Weisskirch (16). This suggests that there may be some external factors (most likely in the social environment) which are required, apart from personality type, to encourage sexting in the study environment. Further research is required in this regard.

Respondents with moderate to severe problematic cell phone use were at least two times likely to indulge in all of the sexting behaviors and were six times likely to ask for such messages, similar to earlier reports (16). This makes moderate to severe problematic phone use to be the strongest predictor of sexting. This finding underscores the need to have more studies looking into how mobile phone use affect young people in the study area as phone possession was very high in this study population. These studies are also necessary so that appropriate interventions which will ensure safe phone use by this population can be instituted. Male sex predicted sending of sext but not receiving of sext and this may be a way of initiating romantic relationships or it may represent expression of masculinity. The older age group were also predictive of sexting and as earlier discussed, this may be due to the higher probability of them being involved in romantic relationships.

The lower prevalence of ever had sex compared with earlier Nigerian studies (31, 32) could be because the study population were schooling young people. Out of school young people have been shown to initiate sex earlier in Nigeria (32). However, the proportion of the study population involved in risky sexual behavior (early sexual debut, non-use of condom at last sexual intercourse, multiple sexual partners, and having sex under the influence of alcohol or drugs) is alarming. The age group studied still has a high prevalence of HIV in Nigeria, higher than the national prevalence rate (33); apart from other untoward consequences of high-risk sex like unwanted pregnancy. This makes it important to strengthen the existing programs to ensure that young people in the study area practice abstinence or safer sex (avoiding multiple sexual partnership, correct and consistent condom use). The only risky sexual behavior associated with both sending and receiving of sext was having multiple sexual partners. This suggests that sexting may predispose to risky sexual behavior or it is being used to maintain risky sexual behaviors. Earlier studies, including longitudinal studies, have shown that there is no consistent pattern of association of sexting with risky sexual behavior (7, 34). The reason for this is not clear which makes qualitative research studies desirable to answer this question. Ever had sex was also associated with sexting which still links sexting with actual sexual activity and so sexting may not just be an alternative to physical sexual activity as earlier suggested (11). This is also strengthened by those who initiated sex shortly after exchanging sexual messages, so sexting can be a primer for physical sexual activity. This is also an avenue for risky sex and its consequences.

There are a number of limitations in this study which can affect the application of the findings to other Nigerian young people. The study was conducted only among young people who are in school. Similar studies among out of school young people in the same area may yield different results. The cross-sectional nature of the study also did not allow causality to be determined and so it is difficult to conclude that sexting was responsible for risky sexual behaviors and problematic phone use and *vice versa*. The data were also self-reported and these may not truly show what the respondents actually do. The desire to appear acceptable may make underreporting of sexual risk behaviors and problematic phone use despite the anonymity that was maintained in the study. Thus, the reported prevalence rates for sexting, risky sexual behaviors, and problematic phone use may be less than the true rates.

In conclusion, this study found that the young people in Ibadan, Nigeria, engage in sexting, and this is found more among males, the LGBT, and those older than 19 years. High extraversion personality and moderate to high problematic phone use were predictive of sexting behavior. The sexually active study participants had a high rate of risky sexual behavior but only having multiple sexual partners and early sexual debut were associated with sexting. Some young people got sexually involved for the first time with the people they exchanged sext with shortly after sexting. Awareness of the existence and dangers of sexting among parents and young people is important to prevent possible problems associated with sexting among this age group. More research about the effect of phone use among young people is also important in the study area as phone possession among them is high. There is a need for qualitative and longitudinal studies to have an in depth understanding of why young people engage in sexting and the context in which sexting is carried out. Such studies can also truly establish causality with the identified associated and predictive factors.

## Ethics Statement

Ethical approval was obtained from the Oyo State Research Ethical Review Committee of the State Ministry of Health, in Ibadan. Permission to conduct the study was obtained from the heads of schools, with written informed consent obtained from students aged 18–24 years in accordance with the Declaration of Helsinki. Students less than 18 years provided assent.

## Author Contributions

OO and FB were involved in the conceptualization of the research, the data collection, analysis, and interpretation. The drafting of the manuscript and approval of the final version was also done by OO and FB.

## Conflict of Interest Statement

The authors declare that the research was conducted in the absence of any commercial or financial relationships that could be construed as a potential conflict of interest.
